# Discovery and characterisation of socially polarised communities on social media

**DOI:** 10.1038/s41598-023-42592-2

**Published:** 2023-09-18

**Authors:** Javier Alvarez-Galvez, Fermin L. Cruz, Jose A. Troyano

**Affiliations:** 1 Department of Biomedicine, Biotechnology and Public Health, University of Cadiz, Avda. Ana de Viya, 52, 11009 Cádiz, Spain; 2 Computational Social Science DataLab (CS2 DataLab), University Research Institute for Sustainable Social Development (INDESS), University of Cádiz, Avda. de La Universidad, 4 (Campus de La Asunción), 11406 Jerez de La Frontera, Spain; 3 Department of Computer Languages and Systems, University of Seville, Avda. Reina Mercedes s/n, 41012 Seville, Spain

**Keywords:** Risk factors, Computational science, Human behaviour

## Abstract

Social polarisation processes have become a central phenomenon for the explanation of population behavioural dynamics in today's societies. Although recent works offer solutions for the detection of polarised political communities in social media, there is still a lack of works that allow an adequate characterization of the specific topics on which these divides between social groups are articulated. Our study aims to discover and characterise antagonistic communities on Twitter based on a method that combines the identification of authorities and textual classifiers around three public debates that have recently produced major controversies: (1) vaccination; (2) climate change; and (3) abortion. The proposed method allows the capture of polarised communities with little effort, requiring only the selection of some terms that characterise the topic and some initial authorities. Our findings show that the processes of social polarisation can vary considerably depending on the subject on which the debates are articulated. Specifically, polarisation manifests more prominently in the realms of vaccination and abortion, whereas this divide is less apparent in the context of climate change.

Community detection on social media has become a promising field of study in recent decades^1^. The gradual rise of the many social media platforms such as Facebook, Twitter or Instagram, and their progressive integration in our daily lives, has increased the scientific interest in defining online communities with structural characteristics and social dynamics that have traditionally been of interest in disciplines such as communication, political science and sociology^2^. While characterization of the structural properties of online communities and studies of their respective behaviour is nothing new within the field of social sciences, these new platforms have aroused the interest of other disciplines such as physics and computer science, which have contributed with new methods for massive data capture and analysis of their complex dynamics^3^. Therefore, the incorporation of innovative ways to analyse and capture social media data has renewed interest in the study of online community formation.

Although the scientific literature offers different approaches for the detection and capture of polarised communities in social media^4–9^, there is still a lack of methods that allow for a comprehensive characterization of the structure of these communities and the subsequent comparison of the specific topics of debate that generate social controversy^9^. Solutions based on signed networks (i.e., those composed of both positive and negative links measured among a set of entities) have proven to be high quality in classification tasks^4, 7^. However, these results have been found to be limited in terms of the explanatory power of the message content^10^. Solutions based on user ratings have also been proposed as a method for the detection of opposing communities on social media platforms, an approach which captures antagonistic groups from their indirect social interactions^8^. However, although these methods take into account the evaluation of information provided by users, they generally do not consider the structure of the relationships between the different users and opinion leaders that make up these online communities^8^. In addition, both methods have limitations that make them unfeasible for the proper detection of antagonistic communities on certain social media platforms^9,11^. Currently, these problems are being tackled through the development of platform-oriented algorithms that, combining both user content information and link networks, improve the quality of the community detection on specific social media^12^.

On the other hand, although in the specialised literature we can find specific works that address polarisation in social media around different issues of interest (e.g., political campaigns, vaccine, global warming, etc.)^13–17^, the need for comparative studies around different conflicting topics that enable a better characterisation of the different communities and a better understanding of the polarisation processes is evident^18^. Recent works have addressed the polarisation processes of communities in the context of the COVID-19 pandemic, although most of these studies are still strongly focused on the analysis of political opinions and governmental actions aimed at controlling the pandemic^19^.

Some descriptive studies are emerging in the context of the current health emergency that provide new findings on both user and topic characterization and may be relevant to understand the polarisation of certain public debates such as vaccination opinions and health behaviours^20,21^. However, there is still a lack of studies that offer a detailed characterization of the topological structure of pro- and anti- communities and their arguments for and against critical societal topics, which is a fundamental step to understand and address misinformation and disinformation in the context of social media platforms^22–24^, i.e., false, erroneous or poor-quality evidence that might increase and reproduce existing social divides. In fact, in the current context of social polarisation due to the misinformation spread by the new social media ecosystem^25^, there is a clear need for new methods to characterise online communities that allow us to develop new strategies to understand the motives behind the opinions and behaviour of the opposing communities, whether in relation to the COVID-19 vaccination debate or other controversial public issues such as climate change, gun control and the anti-abortion debate, all of which may ultimately have a major impact on our social and health systems through people behaviours.

This study aims to discover and characterise antagonistic communities on Twitter based on the identification of authorities and textual classifiers that allow us to compose the network structure of these opposing graphs. To test our algorithm, we focused on three public debates that have sparked major controversy on social media in recent years: (1) vaccination; (2) climate change; and (3) abortion.

## Results

To obtain the communities for each of the topics under study, we started with four seed authorities, two representing each of the positions on the topic (for example, for or against vaccination). An authority is a user who writes a huge number of tweets related to the topic, which, due to their high number of followers, get a high number of impressions in the social network. These initial authorities have been chosen manually, by consulting the timelines of users with a high number of followers (at least 10 K followers) obtained from People Search on Twitter. This way we ensure that the tweets are strongly focused on the topic and their position in the debate is clear, either for or against.

The authorities and all their followers conform the nodes of the initial community graph, so that two nodes are connected by a directed arc if there is a following relationship from one user to another. Although some authors propose using other interactions such as mentions, retweets, or replies^12^, affiliation networks based on following relation are usually more cohesive than interaction networks^26^, since such a relationship generally implies a certain ideological alignment between the users^27^; in addition, we believe that the choice of the following relationship together with the application of a centrality metric is the most appropriate to avoid the selection as authorities of bots and spam accounts.

**Figure 1:**
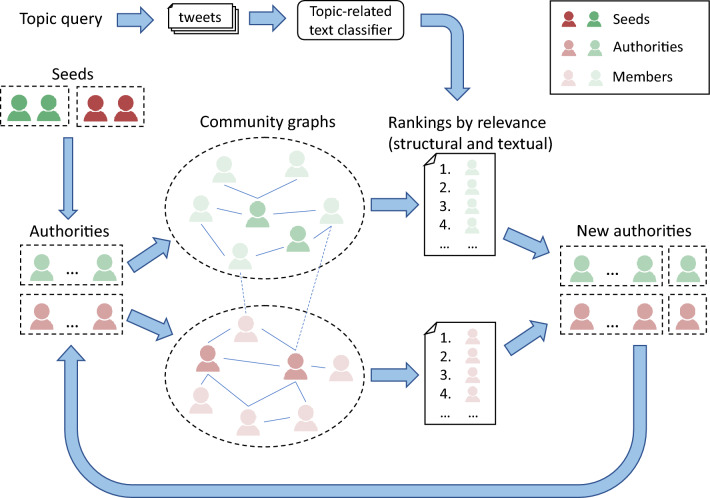
Community extraction process.

Based on the network obtained, we search for new authorities by combining two metrics computed for each user: a structural one, which informs us about the centrality and relevance of the users in the network, and a textual one, which informs us about the closeness to the topic of the users' tweets. For the first metric, we calculate the *PageRank (PR)* score of the nodes of the community graph^28^; while for the second, we use binary text classifiers, which tell us whether or not a tweet is related to the topic. An extended network is obtained by adding followers of the new selected authorities. This process is run iteratively, as shown in Fig. 1. Details on both metrics and how they are combined to select new authorities at each iteration are fully described in the "Methods" section.

### Validation

This section includes a series of experiments to validate the reliability of the community extraction process. These experiments were carried out on the same three target topics we will focus on in the results: (1) vaccination; (2) climate change; and (3) abortion. Our three main objectives are: (a) to measure the accuracy of the classifiers for the three domains; (b) to check that the quality of the new authorities detected does not degrade as the iterations of the process progress; (c) to corroborate that the process converges to equivalent communities even when starting from different seeds, provided that the seeds are sufficiently representative of each community.

#### Accuracy of classifiers

Table 1 shows the search terms used to retrieve the tweets corresponding to the target topic and the accuracy of the classifiers using tenfold cross validation. These classifiers were trained on collections of more than 300 K tweets, 50% corresponding to the target topic, and the remaining 50% extracted from a random sampling of Twitter (see "Methods" section for more details). The results demonstrate a high level of accuracy, indicating that the classifiers have a high degree of reliability and that the topic definitions are well-established from a terminological viewpoint.

**Table 1:** Document classifier evaluation.

Domain	Terms	Accuracy (%)
Abortion	Abortion	93.63
Climate change	Climate change	92.71
Vaccination	Vaccination, vaccine	96.14

#### Closeness of the community to the topic

This experiment seeks to measure the proximity of each community to the different topics under study. To do so, we relied on the text of the tweets written by the most relevant users at each iteration to calculate the *closeness to the topic* indicator. More specifically, we calculated the average of the topic membership values provided by a domain text classifier for the 100 users who obtained the highest *PageRank (PR)* in each step. We used the same text classifiers as were used to calculate the topic proximity metric.

**Figure 2:**
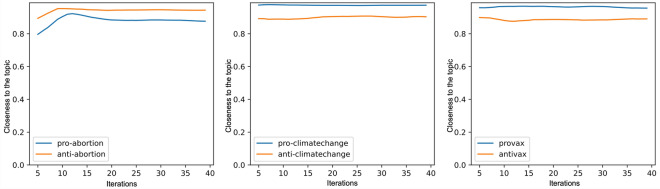
Evolution of closeness to the topic.

Figure 2 shows the evolution of this indicator for the three selected topics. As can be seen, the values in the three thematic areas and for their corresponding pro- and anti-communities are very high. In no case do they drop below 90% and no degradation is observed as the iterations progress. This confirms that the process does not lose its focus on the topic marked by the initial seeds and that it continues to recover new candidates linked to each community in a stable manner.

#### Seed stability

The main input of our method is the small set of authorities selected as seeds. With this experiment, we sought to determine how the choice of seeds affects the subsequent process of authority detection. More specifically, the aim is to evaluate whether the method is capable of converging to similar communities when starting from different seed sets. To this end, two different experiments were carried out for the abortion topic, in both cases using four seeds for each pro- and anti- community. The seed sets of both experiments were completely disassociated. In all cases they were relevant users of each community, but none of the seeds from experiment 2 were used as seeds in experiment 1.

**Figure 3:**
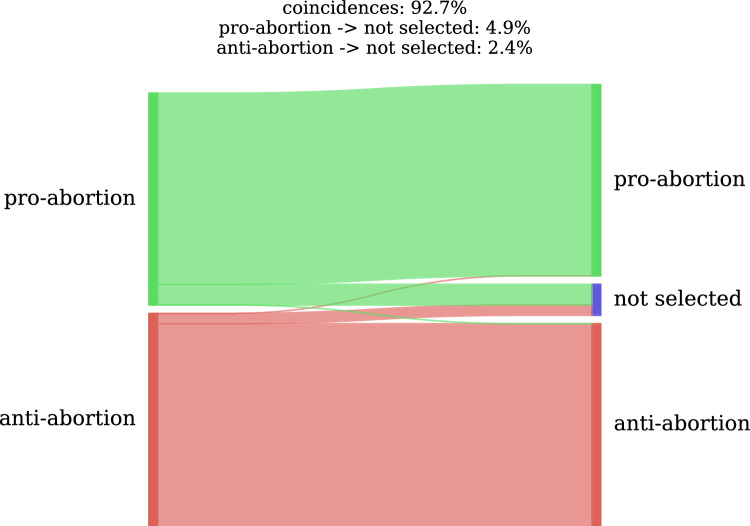
Seed stability for abortion communities.

Figure 3 shows the correspondence of the authorities detected in both experiments using a Sankey diagram. As can be seen, the degree of convergence of the communities is high, even when starting from totally different seed sets. After 80 iterations, 92.7% of the authorities in experiment 1 were also detected in experiment 2. There was only a 7.3% difference between the two experiments, 4.9% in the pro-abortion community and 2.4% in the anti-abortion community.

This experiment demonstrates that our process is stable with respect to the initial seed choice: by choosing representative users from each community as seeds, the process converges to communities that are internally homogeneous even when starting from different seed sets.

#### Network visualisation

**Figure 4:**
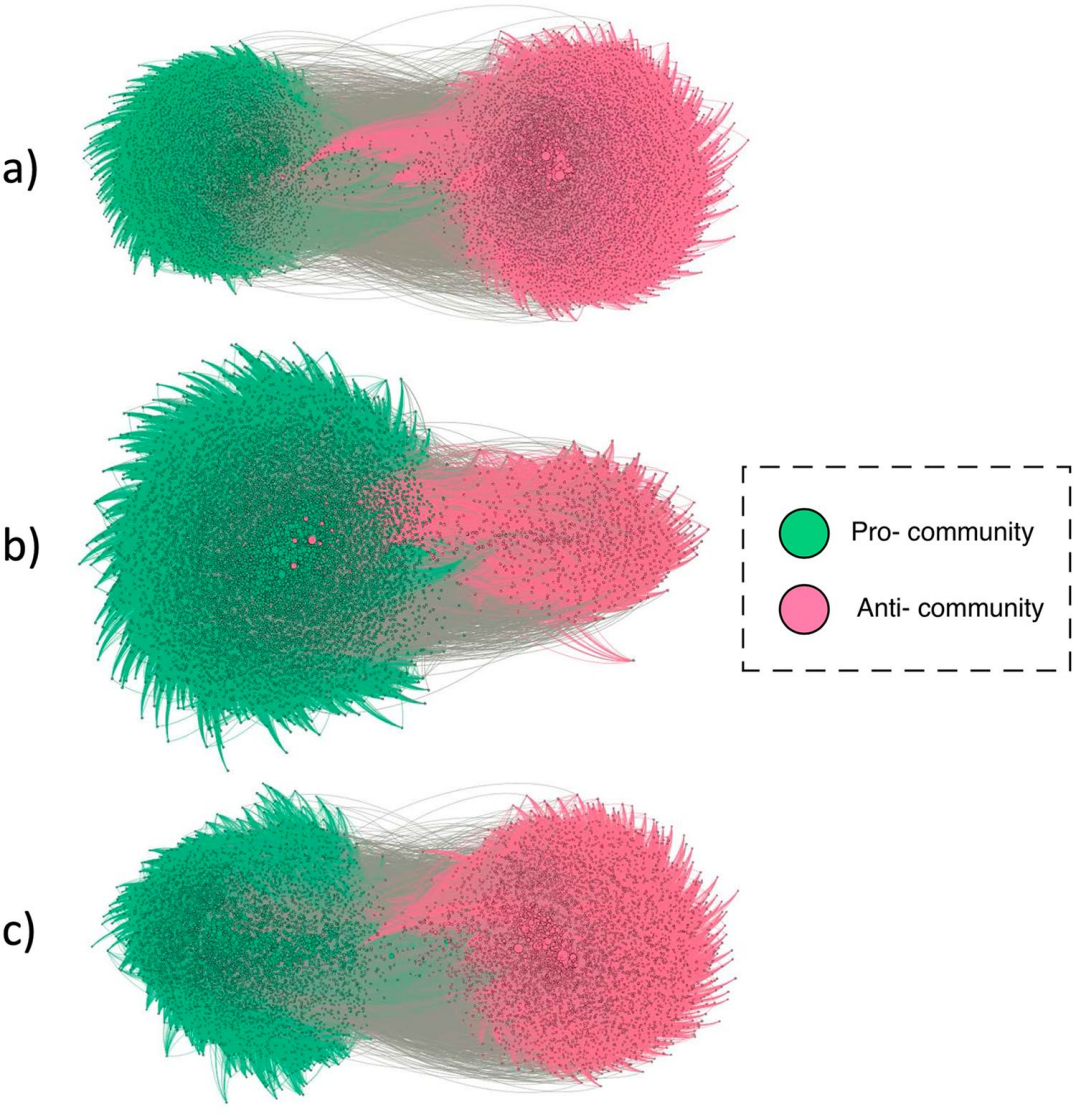
Graph visualisation of the three communities: (**a**) abortion, (**b**) climate change, and (**c**) vaccines. Green is used for “pro-” communities and red for “anti-” communities.

Figure 4 shows a visualisation of the graphs obtained for the three communities. From the three resulting networks for each of the topics analysed, we can see that the abortion and vaccine communities are relatively balanced, while the climate change communities are more unbalanced. Additionally, it is of interest to note the presence of individuals associated with the anti-climate community who possess a high degree of connectivity to the pro-climate community. This is evidenced by the central position in the pro- zone assigned by the proximity display algorithm to those individuals (Fig. 4b).

#### Content analysis

This last experiment validated the quality of our method by analysing the tweets written by the selected authorities during the whole process. We sought to verify that the terms most used by each antagonistic community effectively correspond to pro- and anti- positions.

The tests were performed with the authorities of the three selected topics. For each of the two communities (i.e., pro- and anti- communities) we built a corpus with the last 200 tweets written by the detected authorities in 40 iterations. From these corpora we extracted the most representative keywords to check if they corresponded to terms relating to each community in the domain of the discussion. Keyword extraction was performed using KeyBERT^29^, a tool that uses the embeddings generated by the transformer-based language model BERT^30^ to obtain the words that best capture the semantics of a complete text. Thus, we obtained a list of keywords for each tweet, along with a degree of relevance ranging between 0 and 1. For each term, we calculated their average relevance and relative frequency. They were then filtered to keep the terms with the highest average relevance and relative frequency.

Table 2 shows the 15 most relevant terms of the three thematic communities (i.e., abortion, climate change and vaccines). In the case of the abortion issue, the most frequent terms among pro-abortionists refer to the right to decide in different geographic locations in the USA (those where this issue has been most debated in recent years), while anti-abortionists refer to terms related to the right to life of the unborn (*savethebabyhumans* or *prolife*) and religion (*catholic*). The debate on climate change seems to be divided between the sustainability and resilience discourse of climate change advocates (*adaptation*, *climatecrisis*, *sustainable*, *lossanddamage*, *resilience*, *forest*, *africa*, among other terms), versus the climate change denialists who usually speak of corruption and misinformation from the political and scientific spheres (*corruption*, *climatehoax*, *verifiable*). Finally, as observed, the lists of terms obtained perfectly reflect the lexicon used by each community. The *provax* community includes terms such as *COVAX*, the initiative aimed at equitable access to COVID-19 vaccines, or the term *vaccinneswork*, a digital platform covering news about health and immunisation, were included. On the other hand, the most relevant terms in the *antivax* community include *autism*, *deaths*, *alumninium* (which is widely used as anti-vaccine arguments) and *vaxxed*, the controversial documentary that associates vaccines with autism.

**Table 2:** Top-15 terminology for abortion, climate change and vaccines communities.

Abortion		Climate change		Vaccines	
Pro-abortion	Anti-abortion	Pro-climate change	Anti-climate change	Pro-vaccines	Anti-vaccines
Vote	Prolife	Finance	Lt	Covax	Autism
Clinics	Pregnancy	Adaptation	Globalwarming	Immunization	Vaxxed
Donate	Unborn	Africa	Corruption	Vaccineswork	Vexit
Abortionjustice	Parenthood	New	Canadanews	Diseases	Deaths
Vision4abortion	Womb	Join	Climatehoax	Ebola	Aluminum
Trans	Marchforlife	Climatecrisis	Tim	Vaccinate	Mandatory
Ballot	Preborn	Lossanddamage	Climatologist	Infectious	Masks
Hyde	Savethebabyhumans	Sustainable	Costofnetzero	Misinformation	Pfizer
Indiana	March	Summit	Scorecard	Polio	Pharma
Abortionishealthcare	Prolifeyouth	Netzero	Facts	Globalhealth	Posted
Kansas	Congratulations	Cop26	Weather	Cancer	Trumptwitterteam
Texans	Lifeisahumanright	Resilience	Climatechangeisreal	Pneumonia	Inflammation
Stigma	Catholic	Sustainability	Martyball39	Covidvaccine	Notgoingaway
Voting	Pregnant	Forests	Alberta	Vaxxers	Covidvaccineadverseevents
Abortionisessential	Heartbeat	Pollution	Verifiable	Malaria	Cdcwhistleblower

**Figure 5:**
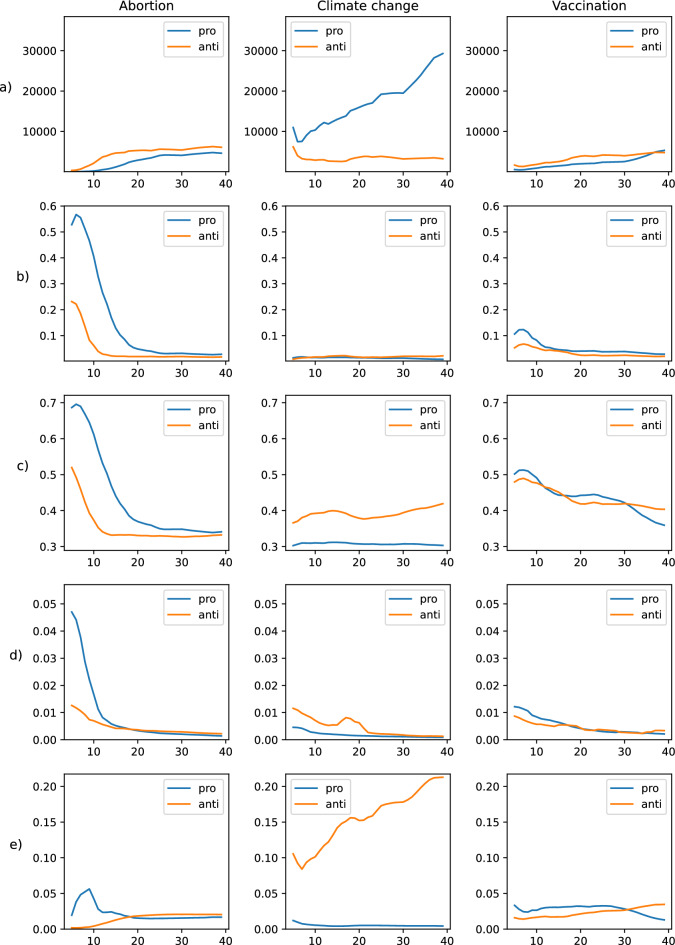
Characterization of intra- and inter-community structure over iterations of the method (x-axis). The y-axis shows: (**a**) Size (nodes); (**b**) Density; (**c**) Reciprocity; (**d**) PageRank of new authorities; (**e**) Edges among communities.

Sometimes relevant words may seem more akin to the opposing community. This is the case of *climatechangeisreal* which is a term that appears as relevant to the *anti-climate change* community. The explanation for this apparent paradox is that these are terms (in this case a hashtag) that are at the centre of a debate, and that are used by the opposing community as a reference in their interventions precisely to refute it. For example, the term *climatechangeisreal* is cited in this way 112 times in the 10,740 tweets analysed from the *anti-climate change* community. This represents a fairly high relative frequency of 1.04%. In the case of the *anti-climate change* community this phenomenon is corroborated by the *edges among communities* metric (Fig. 5e) in which it is clear that members of the *anti-climate change* community are very likely to quote members of the pro-climatechange community in their interventions.

### Experimental results

Following experimental validation of the reliability of our community extraction process, this section shows the results of its application to three selected topics: abortion, climate change and vaccines. For each of these domains 40 iterations were run, starting from only two initial seeds for each community. The runs for each of the above domains were launched on February 4, March 26 and August 17, 2021, respectively, and were spread over a period of between two and three weeks, due to the limitations of using the Twitter API (no breaks were taken between each of the method iterations). In this section we will compare the networks obtained for each anti- and pro- community and interpret the results in terms of the social characteristics of each domain.

This comparative analysis is performed using descriptive metrics of social network analysis on directed graphs obtained for each community. A directed graph is a tuple as defined by Eq. (1).

1 $$G = \left( {V,E} \right)$$

where

- *V* is the set of vertices (or nodes) *v* of the graph, i.e., in our case community users,
- *E* ⊆ {(*u*, *v*) ∈ *V*^2^ |*u* ≠ *v*} is the set of arcs (also links or edges) of the network, in our case follow relationships between users.

#### Size of communities

The size of a community network is defined in Eq. (2) as the number of nodes it contains.

2 $$size\left( G \right) = \left| V \right|$$

Figure 5a shows the evolution of the volume along the iterations for the three domains selected. It can be seen that while the antagonistic communities of the abortion and vaccine domains evolve in a similar manner, the pro-climate change community has a larger size with linear growth in relation to the number of iterations.

#### Graph density

Graph density determines the proportion of edges (i.e., arcs or links) a graph has. In a dense graph, the number of edges is close to the maximum number of possible edges. On the contrary, a sparse graph is a graph with a very low number of edges, i.e., close to the number of edges it would have if it were an empty graph. The density is calculated using Eq. (3) as the ratio between the existing edges and the number of all possible edges.

3 $$density\left( G \right) = \frac{\left| E \right|}{{size\left( G \right)\left( {size\left( G \right) - 1} \right)}}$$

Figure 5b shows the evolution of the density of antagonistic communities for the three selected domains. As can be seen, starting from the initial seeds the pro-abortion community is the one with the highest density in the first iterations. However, this initial high density progressively declines as the method evolves. In the climate change domain, the communities have a low and very similar density. Meanwhile, the density of the pro-vaccine community is somewhat higher than the anti-vaccine community.

#### Reciprocity

Reciprocity is a measure of the probability that vertices of a directed network are mutually linked to each other. The detection of reciprocity patterns may indicate the existence of self-organising tendencies in a community. Reciprocity is calculated with Eq. (4) as the ratio of the number of edges pointing in both directions to the total number of edges.

.

4 $$reciprocity\left( G \right) = \frac{{\left| {\left( {u,v} \right) \in Eif\left( {v,u} \right) \in E} \right|}}{\left| E \right|}$$

Figure 5c shows the evolution of reciprocity for each of the domains (abortion, climate change and vaccines) in the different iterations of our experiments. Greater reciprocity is observed in the pro-abortion, anti-climate change (i.e., climate change denialist groups) and anti-vaccine communities. Firstly, the higher reciprocity of pro-abortion is expected due to the high graph density of the community. Secondly, there is a significant gap in the reciprocity of the antagonistic communities of the climate change domain, with the anti-climate change community showing the highest number of reciprocal ties. Finally, it can be seen that although both antagonistic communities in the vaccine domain show similar reciprocity in the first iterations, the number of reciprocal ties declines earlier in the pro-vaccine community, while in the anti-vaccine community it remains relatively stable.

#### Evolution of PageRank of new authorities

In our community extraction process, *PageRank (PR)* plays a key role in the identification of new authorities, as explained in the "Selection of authorities by relevance" section. PR is a centrality measure of the nodes of a graph with respect to the link structure, so it allows us to score the importance of a user in terms of the impact of its tweets.

We have plotted the evolution of the mean of PR of the new authorities selected in each iteration (Fig. 5d). As expected, the most relevant authorities are captured in the first iterations of the method. As the iterative method progresses, the new authorities detected become less and less relevant. The initial high impact of the authorities of the *pro-abortion* community is interesting, well above the rest of the communities. Also interesting is the small change of trend observed in the *anti-climatechange* community. The latter is a sign that the detection method is adaptive and that for the *anti-climatechange* community, around iteration 15, it has pivoted to detect a more relevant set of authorities than the immediately preceding ones.

#### Edges between communities

This indicator allows us to evaluate the degree of dependence between communities. It can provide us with very valuable information when determining the argumentative leadership in the substantive debate between two antagonistic communities. Our method produces two communities (pro- and anti-) for each domain, which we have modelled using directed graphs (*G*_*pro*_ and *G*_*against*_). However, the possibility exists that users from one community may have follow relationships towards users from another community. The notation *E*_*A*→*B*_ is used here to reflect the set of arcs from users of community *G*_*A*_ to users of community *G*_*B*_.

In order to make this value independent of the dimensions of the communities, we decided to calculate a relative value. Thus, the ratio of arcs between communities is calculated by the Eq. (5) as the proportion of arcs pointing to nodes of the community *G*_*B*_ with respect to the total number of arcs of the nodes of the community *G*_*A*_.

5 $$crossedges\left( {G_{A} ,G_{B} } \right) = \frac{{\left| {E_{A \to B} } \right|}}{{\left| {E_{A} } \right| + \left| {E_{A \to B} } \right|}}$$

As can be seen in Fig. 5e, the anti-climate change community has the highest proportion of arcs pointing to the pro-climate change community. This is also observed in the anti-vaccine and anti-abortion communities as the extraction process evolves, although the differences are not as notable.

## Discussion

The experiments applied to online Twitter debates regarding abortion, climate change and vaccination show that our extraction process offers promising results for the identification of opposing communities based on the initial seed authorities. The findings confirm the validity of our method for the capture and subsequent characterization of antagonistic communities. Additionally, the incremental analysis over the iterations of our method shows important differences in the network metrics depending on the size and topology of the communities we capture. It is important to emphasise that the discussion in this section is based on the evolution of the metrics throughout the execution of the iterations of the method, which tries to capture the target community at a specific point in time; it is therefore not an analysis of the temporal evolution of the communities.

Starting with the pro-vaccine and anti-vaccine communities, we observe that as the data collection process evolves, the results obtained seem to show a greater reciprocity among the members of the anti-vaccine community, as well as a greater following of authorities on this issue despite its lower overall density. In other words, although it may be said that the anti-vaccine community obtained from our method initially has a less compact structure than the pro-vaccine community, anti-vaccine users show greater reciprocity towards the members of their own community^31^. This is also the case for anti-climate change communities (i.e., climate change deniers).

This group shows higher relevance authorities and a greater reciprocity to their own community. The case of abortion is interesting in that these communities have higher relevance authorities and higher density and reciprocity (particularly pro-abortion groups), which could be related to dissemination strategies based on co-hashtagging and retweeting in communities that are relatively closed and immobile in their respective positions^32^. Unlike pro- and anti-vaccine communities, whose polarisation is mainly determined by socioeconomic and ideological differences which, depending on the context, can create a certain movement among vaccine hesitant groups (e.g., economic problems gaining access to vaccination, distrust of new vaccines, pharmaceuticals, interests of governments and health organisations, etc.), pro- and anti-abortion communities are influenced by a more complex interplay of demographic, religious beliefs, ideological, and cultural factors^33,34^. In other words, it could be said that in the case of abortion it makes sense that the opinion leaders of these communities are followed and supported by other members of their own community, but not by other members with totally opposite ways of thinking^34^. This may be a possible explanation for the sudden drop of the trend lines in the indicators mentioned above.

While the results show relatively balanced communities for the vaccination and abortion domains, this is not the case in the climate change groups. In fact, in this experiment there were significant differences between the climate change communities. As with the topics of vaccines and abortion, the climate change debate on social media often takes place in polarising 'echo chambers', but this conversation also takes place in 'open forums', communities of mixed opinions and attitudes that reduce polarisation and stimulate public debate^35^. Thus, due to the greater openness of the debate and the greater variability of the actors involved in this topic, polarisation is less defined in the case of the climate debate as compared to the clear opposition and closeness of communities discussing vaccines or abortion^36^.

Although further work is needed on these differences in the structure of these online communities, this result seems to be related to the fact that the anti-climate change community does not have such a clear structural organisation on social media platforms^35^. Therefore, while the vaccine and abortion conversation show less interaction between the opposing sentiment communities on Twitter, the pro- and anti-climate change communities show more inter-community interactions^22,36^. In fact, recent findings show that direct anti-climate change arguments may be strategically concealed to avoid public confrontations that might affect the commercial interests of companies and/or governments^37,38^, which could be a possible explanation for the central position occupied by some climate change denialist authorities within the community that believes in climate change and promotes actions to combat it (Fig. 4). This hypothesis seems to be supported by previous studies showing that, in general, there is greater support for climate change on Twitter from academia, governments that promote campaigns to combat this problem and news agencies that frequently include this issue in their agenda, resulting in a greater following and support from individual private users^39^. These findings also reinforce the idea of greater openness towards mutual understanding between polarised communities around the climate debate.

In any case, the greater interaction between climate change communities should not lead us to think that the discourse of deniers may have less impact on the ideas and behaviour of climate change supporters. As shown by other studies focusing on misinformation on social media, emotional and anecdotal content is also more likely to be shared on these platforms^23,24^. Similarly, a recent study focusing on polarisation of the vaccine issue has shown that even smaller communities can play a critical role in the Twitter conversation, noting that anti-vaccination groups can easily reach undecided and hesitant groups on social media due to their higher involvement, while pro-vaccination groups have a more peripheral positioning in the conversation^32^. This result shows that minority discourses based on anecdotal evidence and malicious, misleading or erroneous content can be positioned and reproduced through the mainstream narrative.

In addition to obtaining results that fit with the existing evidence, our extraction process has certain methodological advantages. Compared to other social media data capture tools based on keywords or hashtags^4–9^, our method offers: (1) the possibility of extracting and subsequently studying polarised communities built from seed users who are representative agents of the community discourse (i.e., those who are often opinion leaders within their group), especially in antagonistic communities where there is a certain balance between the different poles such as in the abortion and vaccine domains; furthermore (2) it allows extraction of balanced antagonistic communities in terms of sample representativeness with a reduction of the sampling problems associated with online data capture (e.g., occasional discourse related to certain events or selection bias in keywords), which is often an inherent problem of working with massive data from social media platforms; finally (3) it reduces the noise generated by users who intervene in the online conversation but who are not part of the community structure.

In terms of limitations, it should be noted that problems could arise when studying communities that do not present a total polarisation such as those related to climate change, which present a greater inter-community connection and could therefore generate problems of comparison between pro- and anti-communities. Moreover, although our method avoids the selection as authorities of social bots by using a centrality measure (since these users do not usually receive following relationships by genuine authorities), we have not taken into account the inclusion of such social bots as part of the community, which, as shown in recent work could overestimate the metrics of the different communities^40^. In this sense, for future studies it would be necessary to work on the role of social bots within the different communities. In any case, our community extraction method proves useful both for the characterisation of antagonistic communities where the echo chamber effect could occur (those around the vaccine and abortion debates) and for more open communities with less extreme polarisation, which is a fundamental step towards understanding the social dynamics inherent in these diverse communities.

Our study has promising results for future work focused on social dynamics in social media. From a methodological viewpoint, the tool has demonstrated its usefulness and validity for analysis of social polarisation processes. On the one hand, our method is useful because it requires little effort to capture polarised communities around a given topic (i.e., it basically only requires some terms that characterize the topic and the selection of initial authorities based on expert judgement). And, on the other hand, our method is valid in that we are capturing opposing communities, which, based on the findings, fit with what would be expected. Furthermore, our results on the structural comparison of these communities have important implications for social and health communication strategies and could potentially help to reduce disinformation and misinformation on these platforms. Indeed, given the current difficulties faced by government authorities, companies and other organisations to combat hoaxes and fake news, the characterization of these antagonistic communities and their associated dynamics will prove essential to combat this problem in our society.

## Methods

Our community extraction process receives as input two small sets of users (seeds), which may be considered as authorities of the antagonistic communities (pro/anti) of the target topic we are interested in. An authority is a user with a large number of followers and whose posts are mostly focused on the target topic (e.g., vaccines, climate change, etc.). In our experiments, which are described in detail in the "Experimental Results" section, we used only two seeds for each of the antagonistic communities. The process also receives a further input consisting of a set of queries (search terms) that subsequently characterise the selected topic. These queries are used to retrieve tweets on the target topic, in order to train a text classifier which decides whether or not a given tweet corresponds to the target topic.

The process is run iteratively, starting from the seed authorities of each community and obtaining a network by selecting a new authority for each community in each iteration (Fig. 1).

### Construction of the followers’ network

For each of the antagonistic communities a graph is constructed in which the nodes *n*_*i*_ are Twitter users and an edge from *n*_*i*_ to *n*_*j*_ indicates that user *i* follows user *j*. The users selected are the authorities in the community, together with some of the followers of these authorities. To ensure that these followers have a high commitment to the community, they are required to follow a minimum number of authorities (*min*_*followed_auths*_). This threshold is recalculated at each iteration based on the number of authorities selected so far (*n*_*auths*_) using the following formula:

6 $$min_{{followed_{auths} }} \left( {n_{auths} } \right) = \frac{{log\left( {n_{auths} } \right)}}{log\sqrt 2 }$$

This formula ensures that all or most of the authorities are followed in the first iterations, when the number of authorities is small, while relaxing the required proportion of authorities followed as the number of authorities grows. For each of the networks obtained in a given iteration, a new authority is selected. To do so, we rely on two metrics: one related to the structure of the followers’ graph, and the other to the text of the community users’ tweets (proximity to the topic).

### Selection of seed authorities

To locate the seed authorities, we used the Twitter people search; for each target topic we defined a simple query. We used English queries (see terms in Table 2). The use of search terms in English and, therefore, the selection of authorities in that language, are based on the clear predominance of that language at the international level, but, of course, search terms in other languages could be used if we are interested in detecting local communities.

From the users obtained, we selected those with a large number of followers (at least 10 K followers), whose posts were clearly focused on the topic, either for or against. Although an automatic heuristic could be applied to this step, it is convenient to perform it manually, as the small manual effort allows us to make sure that the selected seed authorities are really relevant and good quality ones in their respective communities.

### Selection of authorities by relevance

First, users are ordered according to the *PageRank* of the nodes in the graph^29^, from highest to lowest. A node obtains a higher *PageRank* score if it has many incoming edges, and if these edges come from nodes that in turn have a high *PageRank* score. Therefore, a high score for a node indicates that the user has a high influence in the community: its publications reach many users, either through its direct followers or through the followers of its followers. Multiple studies have demonstrated the effectiveness of PageRank as a measure of centrality in social networks^41–43^.

*PageRank* is an algorithm designed by *Google* to calculate the relevance of any web page on the Internet based on the hyperlinks in which the page is involved. Over time it has become a popular indicator in the field of social network analysis because it provides a reliable and easy-to-compute measure of the global importance of any node in a directed network. The PageRank value of a node is calculated based on the PageRank values of the nodes from which it receives links. The process in Eq. (7) is applied iteratively until the values converge.

7 $$PR\left( N \right) = \left( {1 - d} \right) + d\mathop \sum \limits_{i = 1}^{n} \frac{PR\left( i \right)}{{OE\left( i \right)}}$$

where *N* is the node we want to calculate the PageRank value for, *d* is a damping parameter between 0 and 1, usually 0.85 is used, *n* is the number of nodes in the network, *PR(i)* is the *PageRank* value of node *i*, and *OE(i)* is the total number of outgoing edges from the node *i*. With this formula, each node propagates its relevance (i.e., its PageRank) by distributing it evenly among all the nodes to which it has a link.

### Selection of authorities by topic proximity

Once the PageRank ranking has been obtained, the best ranked user who is not yet an authority is selected as a candidate, and its proximity to the topic is calculated. This value is obtained by applying a text classifier to each of the most recent tweets published by the user (the last 200 tweets are used). The classifier returns a confidence value ranging from 0 to 1, with values close to 1 indicating greater proximity of the text in question to the target topic. We trained a Logistic Regression^44^ estimator on collections of more than 300 K tweets, 50% corresponding to the target topic, and the remaining 50% extracted from a random sampling of Twitter. The tweets corresponding to the target topic were automatically retrieved by querying Twitter using the search terms that the process uses as input to characterise the target topic. We used a bag-of-words, tf-idf representation of the tweets^45^.

The topic proximity metric is obtained as the average of the confidence value of the top 25% of the tweets that received the highest confidence from the classifier. Our aim is that users whose posts cover more than one topic are not necessarily penalised, provided that the target topic is sufficiently represented. If the topic proximity metric is greater than a threshold (experimentally set at 0.98, a very demanding value to ensure that out-of-scope candidates are not selected), the user is selected as a new authority. If not, the next best ranked candidate is selected and the calculation is repeated. Only one new authority is selected from each community in each iteration.

## Data Availability

All data used and analysed during the current study is available from the corresponding author on reasonable request.
